# The presence and stability of nicotine dependence symptoms among adolescents after the implementation of a smoking prevention program

**DOI:** 10.18332/tid/100556

**Published:** 2019-02-04

**Authors:** Monika Csibi, Sándor Csibi, Georges E. Khalil, Zoltán Ábrám, Kristie L. Foley

**Affiliations:** 1 Department of Hygiene, Faculty of Medicine, University of Medicine and Pharmacy Târgu Mureș, Târgu Mureș, Romania; 2 Department of Ethics and Social Sciences, Faculty of Medicine, University of Medicine and Pharmacy Târgu Mureș, Târgu Mureș, Romania; 3 Department of Behavioral Science, MD Anderson Cancer Center, University of Texas, Houston, United States; 4 Department of Implementation Science, Division of Public Health Sciences, Wake Forest School of Medicine, Winston-Salem, United States

**Keywords:** nicotine dependence, school-based smoking prevention, addiction vulnerability, smoking adolescent

## Abstract

**INTRODUCTION:**

Symptoms of nicotine dependence among adolescents occur at an early stage in smoking onset and can be present even with low exposure to cigarettes. We aim to examine the early occurrence of symptoms of nicotine dependence and how they predict later smoking behavior.

**METHODS:**

Participants were ninety-four currently smoking 9th-graders attending high school in Targu Mures, Romania. They were followed for 6 months with two assessment points: baseline, and follow-up at 6 months. We assessed the following: 1) the number of smoked cigarettes in the last 30 days, 7 days, and 24 hours using the Minnesota Smoking Index; 2) vulnerability to addiction manifested in cessation difficulties, using the 9-item version of the Hooked On Nicotine Checklist (HONC), 3) loss of autonomy using the endorsement of at least one HONC item, and 4) dependence, using the modified Fagerström Tolerance Questionnaire (mFTQ). We performed statistical analysis with SPSS version 19, using paired-sample t-tests for comparing the differences between baseline and follow-up data. We also conducted linear regression analysis to demonstrate the predictive role of the assessed variables, such as the scores of the mFTQ and the HONC in maintaining smoking and reported smoking status.

**RESULTS:**

Regression models indicated that baseline-measures for symptoms of dependence (β=0.64, p<0.001), vulnerability to addiction (β=0.47, p<0.001), and loss of autonomy (β=0.34, p<0.001) regarding smoking cessation were significant predictors of smoking, explaining 41.7% of the variability of the reported increase in cigarette consumption. At follow-up at 6-months, the three variables were responsible for 14.9% for the variance in cigarette consumption (R2=0.14, F(1,92)=16.05, p<0.01).

**CONCLUSIONS:**

Nicotine dependence at baseline and at follow-up show significant differences in the control group while in the intervention group the scores remained stable. The findings suggest that participation in the Romanian version of ASPIRE was protective against progression towards nicotine addiction.

## INTRODUCTION

Health care professionals and scientists have emphasized the need to understand the mechanisms of tobacco addiction among adolescent smokers, including the speed with which addiction has led to stable dependence symptoms^1–3^. Few studies have evaluated nicotine addiction and the psychophysiological mechanisms among adolescents^4–6^. The evidence suggests that several symptoms of nicotine dependence occur at an early stage after smoking onset and can be present even if the teenager reports low exposure to cigarettes^1,7^. Early symptoms of dependence can predict the persistence of smoking behaviour, especially in the case of adolescents, suggesting that most symptoms of dependence are strongly predictive of an increased level of tobacco use.^8^

Apelberg et al.^9^ found that 16% of adolescents who used tobacco for one or two days per month reported craving symptoms, with irritability and restlessness during withdrawal reported by 13% of adolescents. Other studies report that early symptoms of addiction are observed among approximately one-third of youths who have smoked 3 or 4 cigarettes and in about 95% of those who have smoked 100 or more cigarettes^10^. Notably, the amount and frequency of the dependency symptoms are not always in concordance; findings suggest that cigarette consumption is more likely to occur after nicotine dependence than before dependence^11^. Gervais et al.^12^ found that among seventh graders, 30% and 20% developed mental and physical symptoms of dependence, respectively, within three months of reporting their first puff. Reports on 6th to 10th graders showed that 25% of new adolescent smokers experienced symptoms of dependence within five months of smoking onset^8,13^. Moreover, difficulty in smoking cessation does not always correspond to the frequency and number of consumed cigarettes^10^. The authors documented that symptoms of early dependence, such as withdrawal, occur even before the daily onset of smoking^10^.

Theoretical approaches of DiFranza et al.^7^ consider the construct of autonomy as a useful explanatory concept of nicotine dependence for adolescents, describing it as observable physiological and psychological difficulties and barriers to smoking cessation. The authors^14,15^ expand the concept of loss of control over tobacco use provided by the Diagnostic and Statistical Manual of Mental Disorders (DSM) through the concept of the loss of autonomy^14,15^, arguing that a smoking teenager whose behaviour and coping methods exhibit severe difficulties in also quitting present a lack of autonomy over tobacco use^14^.

Despite strong evidence of adolescents’ susceptibility to tobacco use and observations regarding their difficulty in cessation processes, the literature treats adolescent nicotine dependence cautiously^16,17^. The occurrence and establishment of the symptoms of addiction among youths remain debated by specialists. A wide range of studies show evidence that tobacco dependence can be present among daily users^4^ and intermittent smokers^13,15,16^. Further, researchers clarify different intervention modalities that support cessation and exclude the risk of addiction^17,18^.

It is still an open research question whether early symptoms of dependence represent a substantial risk for long-term addiction. It is imperative to understand the relationship between self-reported nicotine dependence symptoms and one’s susceptibility to future chronic smoking in order to design effective tobacco use prevention programs for adolescents. A novel aspect of this paper is the examination of the early occurrence of unique symptoms of nicotine dependence, specifically cessation difficulty and autonomy, and how they predict later smoking behaviour. The present study aims to prospectively analyze differences in adolescents’ proclivity toward nicotine use and addiction among participants in a larger study of smoking prevention among adolescents^19^.

For our study, we hypothesized that nicotine dependence scores would remain stable from baseline to the follow-up at 6 months, among participants assigned to the intervention group. Conversely, we hypothesized that reports of nicotine dependence would increase from baseline to follow-up among participants assigned to the control group. We also hypothesised that cessation difficulty and loss of autonomy are positively correlated with the intensity of cigarette smoking at follow-up.

## METHODS

### Setting

In the current study, data were derived from a larger randomized controlled trial of the translated and adapted version of ASPIRE in Romania (‘A Smoking Prevention Interactive Experience’), designed for middle and high school students. This web-based prevention program was originally developed by the MD Anderson Cancer Center and The University of Texas Health Science Center at Houston^20^. It included videos, animations and interactive computer-based activities as well as short films, medical information, and teachers’ and smoking peers’ testimonies. The program aims to provide relevant information on smoking, promote positive attitudes towards tobacco abstinence, and prevent smoking by restructuring the knowledge, attitudes and beliefs related to smoking behaviour. The original ASPIRE program was translated and adapted to Romanian under the name of ASPIRA (‘Activitate Şcolară de Prevenire Interactivă a fumatului în RomâniA’, which means Interactive Smoking Prevention School Activity In Romania)^19^.

### Sample

Data come from a cluster randomized control trial with the aim of testing the interactive smoking prevention program ASPIRA among 1369 ninth-graders (mean age of 14.9 years) in Targu-Mures, Romania, during the 2014–2015 academic year. Of the 1369 adolescents, 94 participants (6.8%) were eligible for the current study, as they were smokers (smoked at least one cigarette in the month preceding baseline) and completed surveys at baseline and at the follow-up at 6 months.

**Figure 1:**
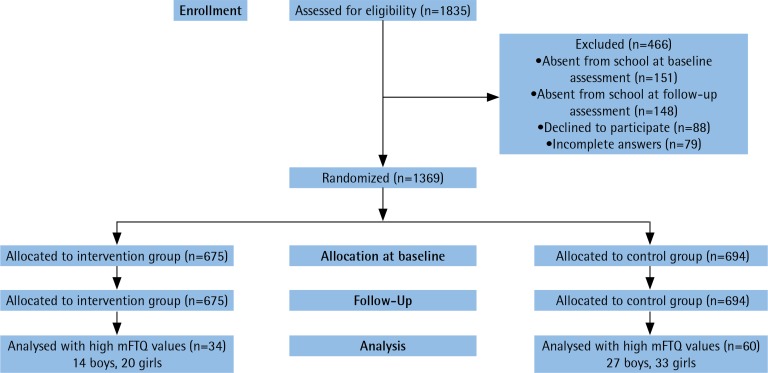
CONSORT diagram with the sample selection of the participant to the study

We summarize the selection procedure in a flow diagram (CONSORT, 2010) that shows the number of participants assessed for eligibility in the initial trial, allocation to baseline and follow-up, and the number of students eligible for the current study (Figure 1). The randomization was conducted for the main trial at the school-level with an allocation to treatment and control conditions one-to-one. The initial sampling frame included all 16 high schools in Tirgu Mures, Romania, with a total of 82 ninth-grade classes^19^.

Participants for the current study are a pool from the trial participants, who are eligible for the current study. Sixty participants were enrolled in the control group, and 34 participants in the intervention group. Altogether, 94 adolescents were included in this secondary data analysis, based on their self-reported smoking intensity and completion of baseline and follow-up testing.

### Measures

The intensity of smoking was characterized based on the Minnesota Smoking Index (MSI)^21^, a composite scale that reflects the number of cigarettes smoked, shown to be highly correlated with biochemical measures among adolescents^22^. For our study, we analyzed the question: ‘Which of the following best describes how often you currently smoke cigarettes?’, with a grading of 1–11, with those graded from 5–11 considered regular smokers (grading: 5=I smoke about 1–3 cigarettes a month; to 11=I smoke more than 1 pack a day)^20^.

We assessed nicotine dependence using a modified Fagerström Tolerance Questionnaire^23,24^, with a

7-item scale that has been psychometrically and biochemically validated among adolescent smokers. As suggested by Prokhorov et al.^25^, we first coded each of the first 7 items from 1 to 4 and the 8th item from 1 to 2. To obtain a final score, the average score of the first 7 items was multiplied by the 8th dichotomous item on morning smoking^25^. The total mFTQ score ranges from 1 to 8. In our study, the mFTQ Cronbach Alpha at baseline was 0.79.

To assess vulnerability to addiction, we used a 9-item version of Hooked On Nicotine Checklist. We omitted an item regarding whether the subject smoked because it was hard to quit, leading to eight items. In previous studies, the internal consistency of the instrument did not change when this item was omitted^26^. To create an indicator variable, we assigned participants a score of 0 if they did not confirm any symptom, and 1 if they confirmed one or more symptoms at each assessment. Cronbach’s Alpha at baseline was 0.74. Loss of autonomy was measured using at least one item from HONC regarding whether the subject smoked because it was hard to quit.

### Statistical analysis

After examining the internal consistency of the psychological tests by calculating the Cronbach Alpha values, we compared baseline and follow-up data concerning our measures using paired t-tests. Further, we conducted a linear regression analysis to demonstrate the predictive role of the assessed variables. We treated as outcome variable the intensity of cigarette smoking (MSI) after the prevention program, and the predictors to be tested were HONC scores, the one-item endorsement of HONC (loss of autonomy in cessation) and mFTQ values (perceived symptoms of dependence). Our stepwise regression model controlled for age and sex. We performed statistical analysis using SPSS version 19 (SPSS Inc.). For baseline comparison of control and intervention groups, we applied a Student’s t-test or chi-squared test for continuous and categorical variables, respectively. A p-value of less than 0.05 was considered statistically significant. The analyzed characteristics, such as age, gender, ethnicity, amount of smoked cigarette, were probabilistically equivalent at baseline.

## RESULTS

At baseline, the mean scores for the modified Fagerström Tolerance Questionnaire (mFTQ) in our sample were 3.06 (SD=1.47) for the intervention group and 2.86 (SD=1.55) for the control group. The Hooked On Nicotine Checklist (HONC) mean values were 3.41 (SD=2.32) for the intervention group and 3.53 (SD=2.41) for the control group. At baseline, the mean value of the reported amount of smoked cigarette was about a pack (20 cigarettes) a week (Table 1).

**Table 1:** Baseline comparison of control and intervention groups of adolescents, Romania in 2017 (n=94 )

*Variables*	*Control (N=60 )*	*Intervention (N=34 )*	*p*
Age, mean (SD)	14.93 (0.516)	15 (0.550)	0.448
Gender, n (%)			
Female	33 (55)	20 (58.8)	0.886
Male	27 (45)	14 (41.2)	
Ethnicity, n (%)			
Romanian	38 (63.3)	21 (61.8)	0.526
Non-Romanian	22 (36.7)	13 (38.2)	
Academic achievement, n (%)			
High grades	33 (55)	27 (79.4)	0.032
Low grades	27 (45)	7 (20.6)	
Amount smoked, n (%)			
Part to 1–3 cig/month	10 (16.7)	5 (14.7)	0.804
1 cig/week to >pack/day	50 (83.3)	29 (85.3)	
mFTQ (SD)	2.86 (1.52)	3.06 (1.47)	0.622
HONC (SD)	3.53 (2.41)	3.41 (2.32)	0.812

First, we conducted paired sample t-tests to inspect differences in our variables between baseline and follow-up, separately for the intervention and control groups. In the intervention group, the obtained scores for the assessed variables showed no significant change over time. Among participants in the control group, we found a significant increase in nicotine dependence (t=-3.18, p=0.002), but no change over time in vulnerability to addiction or loss of autonomy.

In the stepwise regression model noted in Table 2, we introduced vulnerability to addiction, dependence, and loss of autonomy as predictors of the variability of smoking intensity. According to the model, at baseline, dependence explained 41.7% of the variability of reported increase in cigarette consumption (R^2^=0.41, F(1,92)=12.15, p<0.01), from which vulnerability to addiction explained 10.5% (β=0.47, p<0.001), loss of autonomy (measured with at least one item endorsement of HONC) explained 11.7 % (β=0.34, p<0.001), and the nicotine dependence as measured with mFTQ explained 19.5 % (β=0.64, p<0.001) of the variability in smoking intensity reported at baseline. At follow-up and as noted in Table 3, the mFTQ items explained 4.2% (β=0.38, p<0.001), the HONC 5.8% (β=0.32, p<0.001) and the one item endorsement of HONC conceptualized as loss of autonomy 4.4 % (β=0.21, p=0.042) the variability in smoking intensity, manifested in a number of smoked cigarettes six months later. Together, the three variables were responsible for 14.9% for the variance (R^2^=0.14, F(1,92)=16.05, p<0.01) in cigarette consumption reported at follow-up.

**Table 2:** The prediction role (linear regression) of Hooked on Nicotine Checklist (HONC), modified Fagerström Tolerance Questionnaire (mFTQ), and loss of autonomy (at least one item endorsement from HONC) at baseline for the amount of consumed cigarette among adolescents, Romania in 2017 (n=94 )

*Model (steps)*	*Predictors (at baseline)*	*R*	*R^2^*	*SEE*	*FC*	*SFC*
1	Endorsement	0.342	0.117	1.484	12.158	0.001
2	HONC	0.471	0.222	1.393	26.249	0.000
3	mFTQ	0.646	0.417	1.206	65.736	0.000

Dependent Variable: Amount of consumed cigarette at baseline. SEE: standard error of the estimate, FC: F Change, SFC: Significance of F Change.

**Table 3:** The prediction role of (linear regression) smoking status predicted by Hooked on Nicotine Checklist (HONC), modified Fagerström Tolerance Questionnaire (mFTQ), and loss of autonomy (at least one item endorsement from HONC) at follow-up for the amount of consumed cigarette among adolescents, Romania in 2017 (n=94 )

*Model (steps)*	*Predictors (at follow-up)*	*R*	*R^2^*	*SEE*	*FC*	*SFC*
1	Endorsement	0.211	0.044	1.598	4.273	0.042
2	HONC	0.319	0.102	1.549	10.439	0.002
3	mFTQ	0.385	0.149	1.508	16.050	0.000

Dependent Variable: Amount of consumed cigarette at follow-up. SEE: standard error of the estimate, FC: F Change, SFC: Significance of F Change.

## DISCUSSION

Participants allocated to the intervention group reported unchanged scores on nicotine dependence from baseline to follow-up, as we hypothesized. These results suggest stability in smoking cessation-related difficulties from baseline to six months later, despite exposure to the intervention. The t-test results showed no significant increase in loss of autonomy among control group participants, but we observed a significant increase in nicotine dependence scores.

In a study by Kleinjan et al.^27^, different subtypes of nicotine dependence were examined, suggesting individual variation in the presence and severity of dependence symptoms during adolescence. The results indicate that among intervention group participants, perceptions about their smoking-related symptoms might have altered under the recognition of symptoms that they might not have been aware before or by the emotional depth in which the severity of the problem was presented, thus leading to reduced cessation attempts. Recent work of Prokhorov et al.^25^ revealed that the perceived entertainment and the interactivity of the original ASPIRE significantly influence the intention to quit among adolescents^25^. Our results align with those in other literature that provided evidence concerning the increase in the reported dependence symptoms when interventions are lacking^7,14^. Other researchers have also found that adolescents often misperceive dependence symptoms or difficulties in cessation; adolescents with varied experiences with tobacco respond positively to questions related to tobacco addiction. These findings suggest that teenagers, who may find themselves addicted, perceive in a different way the severity of a certain addiction item in a survey and hence their responses to validated surveys can vary^7,14,16,28^. According to DiFranza et al.^7^, even youth who never smoked affirmed their need for a cigarette. Okoli et al.^28^ affirm that the feeling of mental or physical addiction symptoms at such an early age is not necessarily a misunderstanding or measurement error, but instead an indication of tobacco use vulnerability.

Our second hypothesis tested if the variation in nicotine dependence scores was dependent upon the number of cigarettes consumed. The baseline regression model demonstrated a stronger correlation between frequency of smoked cigarettes and dependence. However, the finding was attenuated at follow-up. Thus, our results only partially support our original hypothesis related to consumption frequency and dependence.

Our results support the sensitivity of the HONC and the mFTQ to detect nicotine dependence among adolescent smokers, consistent with prior research^14^. These findings suggest that physical symptoms, such as cravings, may precede the consciousness of the psychological components of tobacco addiction.

The current study offers important contributions to our understanding of nicotine dependence among adolescent smokers. Prevention programs targeting adolescents have been challenged regarding their efficacy for sustained abstinence. One of the most critical issues is identifying the most appropriate individuals to engage in smoking cessation programs, identifying the population for whom primary prevention would be more appropriate, and separating those who are and those who are not already exhibiting symptoms of addiction. There may be value in having tailored interventions that take into account smoking dependence that manifests differently according to different physical, emotional, or social symptoms. A tailored program may also be able to differentiate between vulnerable adolescents at greatest risk of nicotine dependency and those more likely to experiment, but not become addicted.

### Limitations

While this study contributes to the literature on nicotine dependence among adolescents, there are some limitations. The main limitation is the small sample size. Other limitations include a lack of qualitative differences among addicted smokers that could further enhance our understanding of dependence experiences. Moreover, we did not obtain objective measures of nicotine dependence (e.g. cotinine samples) and relied on self-reported symptoms, thereby increasing the risk of socially desirable responses^29^.

## CONCLUSIONS

We conclude that nicotine dependence significantly worsened among adolescents in the control group, while those in the intervention group remained stable. The findings suggest that participation in the Romanian version of ASPIRE was protective against progression towards nicotine dependence. In our study, nicotine dependence as measured by the modified Fagerström Tolerance Questionnaire was a significant predictor of continued smoking. We also demonstrated that the HONC and the mFTG were sensitive predictors of dependence among an adolescent population.

## CONFLICTS OF INTEREST

Authors have completed and submitted the ICMJE Form for Disclosure of Potential Conflicts of Interest and none was reported.
